# Standardized regression-based clinical change score cutoffs for normal pressure hydrocephalus

**DOI:** 10.1186/s12883-020-01719-y

**Published:** 2020-04-16

**Authors:** Alexander Davis, Sevil Yasar, Iris Emerman, Seema Gulyani, Kristina Khingelova, Aruna Rao, Lacie Manthripragada, Mark Luciano, Abhay Moghekar

**Affiliations:** 1 Department of Neurology, Johns Hopkins University School of Medicine, 5200 Eastern Ave CTR STE 5100, Baltimore, MD 21224 USA; 2 Department of Medicine, Johns Hopkins University School of Medicine, Baltimore, MD USA; 3 Department of Neurosurgery, Johns Hopkins University, Baltimore, MD USA

**Keywords:** Normal pressure hydrocephalus, Clinical Change, Tests and measurements, Gait, Cognition

## Abstract

**Background:**

Presently, for patients presenting with suspected Normal Pressure Hydrocephalus (NPH) who undergo temporary drainage of cerebrospinal fluid (CSF) there is no defined model to differentiate chance improvement form clinical significance change at the individual patient level. To address this lack of information we computed standard regression based clinical change models for the 10 Meter Walk Test, Timed Up & Go, Dual Timed Up & Go, 6-Minute Walk Test, Mini-Balance Evaluation Systems Test, Montreal Cognitive Assessment, and Symbol Digit Modalities using data from patients with suspected NPH that underwent temporary drainage of CSF. These clinically significant change modes can classify clinically significant improvement following temporary drainage of CSF at the individual patient level. This allows for physicians to differentiate a clinically significant improvement in symptoms from chance improvement.

**Methods:**

Data was collected from 323 patients, over the age of 60, with suspected NPH that underwent temporary drainage of CSF with corresponding gait and cognitive testing. McSweeney Standardized Regression Based Clinical Change Models were computed for standard gait and cognitive measures: Timed Up & Go, Dual Timed Up & Go, 10 Meter Walk Test, MiniBESTest, 6-Minute Walk Test, Montreal Cognitive Assessment, and Symbol Digit Modalities Test. To assess the discriminate validity of the measures we used correlations, Chi^2^, and regression analyses.

**Results:**

The clinical change models explained 69–91.8% of the variability in post-drain performance (*p* <  0.001). As patient scores became more impaired, the percent change required for improvement to be clinically significant increased for all measures. We found that the measures were not discriminate, the Timed Up & Go was highly related to the 10 Meter Walk Test (*r* = 0.85, *R*^*2*^ = 0.769–0.738, *p* <  0.001), MiniBESTest (*r* = − 0.67, *R*^*2*^ = 0.589–0.734, *p* <  0.001), and 6 Minute Walk Test (*r* = − 0.77, *R*^*2*^ = 0.71–0.734, *p* <  0.001).

**Conclusion:**

Standardized Regression Based Clinically Significant Change Models allow for physicians to use an evidence-based approach to differentiate clinically significant change from chance improvement at the individual patient level. The Timed Up & Go was shown to be predictive of detailed measures of gait velocity, balance, and endurance.

## Background

Idiopathic Normal Pressure Hydrocephalus (NPH) is a neurological condition caused by enlarged communicating ventricles, defined by an Evans index (EI) of at least 0.3 [1, 2]. The classic triad of NPH is gait dysfunction, cognitive decline, and urinary incontinence, with gait dysfunction being an essential symptom of the triad [3–5]. One way to assess if a patient will benefit from a shunt is measuring their responsiveness to temporary drainage of cerebrospinal fluid (CSF), either by a lumbar puncture (30–50 ml) or extended lumbar drainage (300–400 ml) [5]. The goal of temporary drainage of CSF it to compare the results of pre-drain and post-drain objective testing to determine if the patient had a meaningful improvement in symptoms [6].

Assessing change in objective gait or cognitive measures following temporary CSF drainage requires a defined model to ensure that patients are selected for shunt surgery in a reliable and valid manner. Grading scales are the primary models used to assess change in score following temporary CSF drainage. Grading scales are efficient for defining improvement at the group level. However, they have not been validated to differentiate a significant change from chance improvement at the individual patient level [7, 8]. The present study aimed to address this gap of knowledge by establishing standardized clinical change models using data from patients evaluated for suspected NPH to differentiate chance improvement from a clinically significant change at the individual level. Clinically significant change is defined as a change in score on an established clinical measure that is beyond what would be predicted by regression while accounting for measurement error [9]. We will present clinical change models for the 10 Meter Walk Test [10], Timed Up & Go [11–13], Dual Timed Up & Go [14, 15], 6-Minute Walk Test [16–18], Mini-Balance Evaluation Systems Test [19], Montreal Cognitive Assessment [20, 21], and Symbol Digit Modalities [22]. Additionally, we plan to assess the discriminate validity of measures used in patients presenting with suspected NPH designed to assess the domains of gait velocity, balance, and endurance.

## Methods

### Participants

A retrospective chart review of 323 patients who underwent temporary drainage of CSF at the Johns Hopkins Center for CSF Disorders. All patients included in the study were over the age of 60 and seen within the departments of Neurosurgery and Neurology, between October 2013 and March 2019. Patients were included in the study if they had cerebral ventriculomegaly and the presence of gait, cognitive, or urinary dysfunction. For this analysis, we did not classify patients as idiopathic NPH as the same criteria are used to assess change in score for both idiopathic and secondary NPH. However, patients that had a brain tumor or subarachnoid hemorrhage were excluded. The study was conducted with the approval of the Johns Hopkins Institutional Review Board. Since this was a retrospective study involving only data extraction and analysis, informed consent was waived by the IRB. Data once extracted was anonymized for analysis.

### Outcome measures

Physical therapists completed all gait assessments, while trained research assistants completed cognitive testing. Gait assessments were administered in hallways with smooth floors. Assistive devices were used when required for safe ambulation. Patients were required to use the same assistive device for all trials. Pre-drain assessments were completed between 1 and 4 h of the beginning of the drain. Post-drain assessments began when the patient was medically cleared to walk after the drainage procedure, less than 60 min after LP, and between 1 and 4 h after ELD. Gait measures were divided into three domains - velocity, balance, and endurance.

#### Gait velocity

For all gait velocity measures, patients were instructed to walk, “As quickly as you can, safely.” 10 Meter Walk Test (10 MWT): patients started standing up, walked 10 meters in a straight line, and stopped. Timed Up & Go (TUG): patients started seated in a chair with armrests, stood up, walked 10 feet, turned 180 degrees; walked back and sat down in the chair. Dual TUG: identical to the TUG except while patients walked, they performed a serial subtraction of three. All gait velocity measures used the time required to complete measured to hundredths of a second as the final score.

#### Endurance

6-Minute Walk Test (6MWT): Patients walked as far as they were capable of in 6 minutes. The final score for the 6MWT was total distance walked measured in feet.

#### Balance

Mini-Balance Evaluation Systems Test (Mini-BEST): is a dynamic measure of balance consisting of 14 items all scored from 0 to 2. Patients attempted tasks such as rising from a chair with their arms crossed, attempting to raise their heels off the ground while standing upright, etc. The maximum score was 28 points with a higher total score indicating better balance.

#### Cognition

Montreal Cognitive Assessment (MoCA): assesses global cognitive function. The MoCA is scored out of 30 points with higher scores indicating better performance.

Symbol Digit Modalities Test (SDMT): is a test of visuomotor coordination relying on a combination of attention, processing speed and working memory.

### Statistical analyses

Before defining the data analysis plan, we knew that we had attempted temporary drains on patients with extreme levels of impairment that go beyond what would be consistent with suspected NPH. To address this issue, outliers were removed based on pre-drain scores using the Standard Outlier Formula to trim the data [23]. The MoCA lower bound was 6.5 with an upper bound greater than 30 (removed *n* = 6). The TUG lower bound was below zero seconds and the upper bound was 51.37 s (removed *n* = 33). The Dual TUG lower bound was below zero seconds with an upper bound of 77.83 s (removed *n* = 17). Ten MWT lower bound was below zero seconds, with an upper bound of 33.1 s (removed *n* = 31). No outliers were removed for the SDMT, MiniBEST, or 6MWT.

In a sensitivity analysis to assess if there was a significant difference between LPs and ELDs, we compared Pre-drain scores, Post-drain scores, and percent change of scores using t-tests. After removing outliers, there was no significant difference between LPs and ELDs for the MoCA, TUG, Dual TUG, Mini-BEST and 10 MWT. There was a significant difference between groups at pre-drain for the 6 MWT. For patients with a baseline of over 500 ft, there was no significant difference in response to temporary drainage for the 6MWT. For this study, LPs (*n* = 238) and ELDs (*n* = 72) were pooled together for analysis. If a patient had undergone multiple temporary drains, only the first drain was used for analysis.

The primary objective of data analysis was to create empirical models for discerning clinically significant change from chance improvement at the individual patient level. Clinically significant change is defined as a change in score on an established clinical measure that is beyond what would be predicted by regression while accounting for measurement error. To create clinical change models, we used the Standardized Regression-Based model (SRB). We chose the SRB model because it best fits the needs of our population because of the wide range of scores at baseline [24]. For calculating the SRB models, we followed procedures described by McSweeney et al., [9] SRB models can use multiple linear regression to predict the posttest score for an individual based on the pretest score and other relevant variables. The predictor variables used in our regression equations were, Pre-drain score, sex, age, BMI, height, Evans index, past medical history of conditions affecting gait, assistive device used, education level, and depression. However, as others have noted, even when significant, the added predictor variables beyond the Pre-drain (baseline) score did not add any increased predictive value to the SRB models [24–29]. The final models used for all measures were simple SRB models. Once the predicted score has been calculated using the linear regression model, the score can be converted into a change z-score using the following equation: z-score = (Yo -Yp)/SEest, Yo is the observed posttest score, Yp is the predicted score and SEest is the standard error of the estimate from the regression equation. For change to be clinically significant, the z-score must exceed ±1.64 (90% confidence interval) [30, 31].

The secondary objective was to assess the discriminate validity of dividing the gait measures by the domains of velocity, balance, and endurance. To assess if there was a relationship between measures, a correlation matrix using the Pre-drain scores was computed. For measures that were highly correlated (R > 0.70), a Pearson chi^2^ analysis using the outcome of the temporary drain for each measure was performed (outliers were not removed for the chi^2^ analysis). For any combination of measures for which chi^2^ was significant, a regression analysis was performed to assess the relationship between the Pre-drain to Pre-drain and Post-drain to Post-drain scores of the measures. If the Pre-drain regression analysis was significant (*p* <  0.05), a Post-drain regression analysis was performed. For the regression analyses, models were checked for a quadratic or cubic relationship, and heteroscedasticity. If a model was found to be heteroscedastic, cooks’ distance was used with a cutoff of 4/n as a cutoff for overly influential points [32].

## Results

### Participants

Table 1 presents baseline patient demographic data and clinical characteristics including age, race, sex, education, EI, and past medical history that could affect gait (stroke, transient ischemic attack, Parkinson’s disease, peripheral neuropathy, osteoarthritis, degenerative joint disease, and spinal disorders).

**Table 1:** Baseline demographics and clinical characteristics of (*N* = 323) study participants

	Mean (SD) or N (%) *N* = 323
Age (years)	74.88 (6.41)
Sex (male)	194 (60)
Race (Caucasians)	287 (89)
Education (years)	
≤ 12	100 (31)
13–16	120 (37)
> 16	103 (32)
Evans Index (EI)	0.37 (0.04)
Height (meters)	1.69 (0.11)
Body Mass Index (BMI)	27.75 (4.56)
Assistive Device	
None	132 (41)
Cane	89 (28)
Walker	102 (32)
Past Medical History	279 (55)

Past Medical History positive for any of the following: stroke, transient ischemic attack, Parkinson’s disease, spinal disorders, degenerative joint disease, neuropathy or osteoarthritis

Patients were predominantly Caucasian (89%) and highly educated. The mean age was 74.9 years (SD ± 6.4), with a mean body mass index (BMI) of 27.75 (SD ± 4.6), 39% of patients used an assistive device, and 55% of patients had a past medical history that could affect their gait.

### Outcome measures

Table 2 shows Pre-drain and Post-drain scores for the outcome measures.

**Table 2:** Pre-and Post-drain results of cognition, gait, balance, and endurance

	Pre-drain (SD)	Post-drain Mean (SD)	Pearson R
**MoCA (points)**	20.49 (5.05)	21.10 (4.88)	0.83
**SDMT (Points)**	21.72 (9.41)	22.50 (9.55)	0.88
**TUG (sec)**	18.64 (9.72)	15.94 (8.45)	0.91
**Dual TUG (sec)**	27.03 (16.26)	22.57 (12.94)	0.89
**10 MWT (sec)**	12.99 (5.78)	10.84 (4.50)	0.85
**Mini-BEST (points)**	14.70 (5.26)	17.52 (5.20)	0.89
**6 MWT (feet)**	792.45 (446.87)	918.02 (481.71)	0.96

*MoCA* Montreal Cognitive Assessment, *SDMT* Symbol Digit Modalities Test, *TUG* Timed Up & Go, *Dual TUG* Dual Timed Up & Go, *10 MWT* 10 Meter Walk Test, *Mini-BEST* Mini-Balance Evaluation Systems test, *6 MWT* 6-Minute Walk test

The test-retest reliability of the Pre- and Post-drain measures was high, with a reliability coefficient ranging between 0.83–0.96. The percent change of the mean value between Pre-drain and Post-drain gait measures ranged from 14.48 to 19.18%. For the MoCA, the mean Pre-drain and Post-drain percent change was only 2.98%.

Table 3 shows the SRB equations predicting Post-drain scores using the Pre-drain score for each measure.

**Table 3:** Regression Coefficients for Standardized Regression-Based (SRB) models of cognition, gait, balance, and endurance

	ß^a^	C^b^	SE^c^	R-Squared	*p*
**MoCA (points)** **(*N*** **= 221)**	0.800	4.720	± 0.764	0.690	< 0.001
**SDMT (points)** **(*N*** **= 205)**	0.898	2.886	± 0.822	0.770	< 0.001
**TUG (sec)** **(*N*** **= 277)**	0.787	1.273	± 0.458	0.826	< 0.001
**Dual TUG (sec)** **(*N*** **= 275)**	0.705	3.414	± 0.697	0.789	< 0.001
**10 MWT (sec)** **(*N*** **= 273)**	0.661	2.245	± 0353	0.724	< 0.001
**Mini-BEST (points)** **(*N*** **= 290)**	0.880	4.564	± 0.406	0.799	< 0.001
**6 MWT (feet)** **(*N*** **= 306)**	1.031	99.887	± 16.153	0.918	< 0.001

*MoCA* Montreal Cognitive Assessment, *SDMT* Symbol Digit Modalities Test, *TUG* Timed Up & Go, *Dual TUG* Dual Timed Up & Go, *10 MWT* 10 Meter Walk test, *Mini-BEST* Mini-Balance Evaluation Systems test, *6 MWT* 6-Minute Walk test

^a^Beta (slope)

^b^Constant

^c^Standard error of the estimate

Across all measures, patients with minor impairment at Pre-drain required a smaller percent change to reach the threshold of clinically significant change than patients with moderate to high levels of impairment.

For assessing discriminate validity, we found that all baseline gait measures were highly correlated (*r* = 0.67–0.85) (Table 4).

**Table 4:** Discriminate validity Pre-drain correlations of cognition, gait, balance, and endurance

	MoCA	TUG	10 MWT	Mini-BEST	6 MWT
**MoCA (points)**	1				
**TUG (sec)**	− 0.33***	1			
**10 MWT (sec)**	−0.30***	0.85***	1		
**Mini-BEST (points)**	0.39***	−0.67***	−0.68***	1	
**6 MWT (feet)**	0.33***	−0.77***	−0.80***	−.72***	1

*MoCA* Montreal Cognitive Assessment, *TUG* Timed Up & Go, *10 MWT* 10 Meter Walk test, *Mini-BEST* Mini-Balance Evaluation Systems test, *6 MWT* 6-Minute Walk test

*p* <  0.05 *

*p* <  0.01 **

*p* <  0.001 ***

For the chi^2^ analysis, we found the TUG was significantly associated with all other gait measures (6MWT *P* <  0.001; 10 MWT *P* <  0.001; Mini-BEST *P* = 0.023). When applying regressions models using TUG as the predictor variable, there was a quadratic relationship with all other dependent variables.

Table 5 shows for Pre-drain regressions of TUG, Mini-BEST (C = 27.278, ß_TUG Pre-drain_ = − 0.902, ß_TUG Pre-drain_^2^ = 0.011, SE ± 1.096, R-squared = 0.553, *P* <  0.001) and 6 MWT (C = 2089.879, ß_TUG Pre-drain_ = − 95.028, ß_TUG Pre-drain_^2^ = 1.246, SE + 70.744, R-squared = 0.71, *P* <  0.001) were both highly significant and homoscedastic.

**Table 5:** Discriminate validity regression coefficients for the timed up & go at pre-drain and post-drain

	ß^a^	ß^b^	C^c^	SE^d^	R-Squared	*p*
**MiniBEST Pre-drain (*N*** **= 254)**	−0.902	0.011	27.278	± 1.096	0.553	< 0.001
**6 MWT Pre-drain (*N*** **= 264)**	−95.028	1.246	2089.879	± 70.744	0.71	< 0.001
**10 MWT Pre-drain (*N*** **= 242)**	0.782	0.007	0.974	± 0.798	0.769	< 0.001
**MiniBEST Post-drain (*N*** **= 265)**	−0.854	0.009	28.679	± 0.799	0.589	< 0.001
**6 MWT Post-drain (N = 264)**	−116.008	1.651	2307.064	± 66.163	0.734	< 0.001
**10 MWT Post-drain (*N*** **= 243)**	0.441	0.001	3.606	± 0.375	0.738	< 0.001

*Mini-BEST* Mini-Balance Evaluation Systems test, *6 MWT* 6-Minute Walk test, *10 MWT* 10 Meter Walk test

^a^Beta Timed UP & GO

^b^Beta Timed UP & GO Squared

^c^Constant

^d^Standard error of the estimate

The 10 MWT was also highly significant at pre-drain (C = .974, ß_TUG Pre-drain_ = 0.782, ß_TUG Pre-drain_^2^ = − 0.007, SE ± 0.798, R-squared = .769, *P* <  0.001) but heteroscedastic after influential points were removed. For Post-drain regressions of TUG, the Mini-BEST (C = 28.679, ß_TUG Post-drain_ = − 0.854, ß_TUG Post-drain_^2^ = 0.009, SE ± 0.799, R-squared = 0.589, *P* <  0.001) and 6 MWT (C = 2307.064, ß_TUG Post-drain_ = − 116.008, ß_TUG Post-drain_^2^ = 1.651, SE ± 66.163, R-squared = 0.734, *P* <  0.001) were both highly significant. After influential points were removed both analyses were homoscedastic. The 10 MWT was highly significant (C = 3.606, ß_TUG Post-drain_ = 0.441, ß_TUG Post-drain_^2^ = 0.001, SE ± 0.375, R-squared = 0.738, *P* <  0.001) at Post-drain and heteroscedastic after influential points were removed.

## Discussion

In our study, we have created SRB clinical change models for assessing clinically significant change for patients undergoing CSF drainage as part of an evaluation for suspected NPH for the TUG, Dual TUG, 10 MWT, Mini-BEST, 6MWT, MoCA, and SDMT. Clinically significant change is defined as change on an established clinical measure that is beyond what would be predicted by regression while accounting for measurement error. Clinical change models can differentiate clinically significant change from chance improvement at the individual patient level. The most novel finding was that the percent change required for clinically significant change is not fixed across the range of impairment. The percent change in improvement increases as patients become more impaired. For the tests of discriminate validity, we found that the TUG is significantly related to both pre-drain and post-drain measures of the Mini-BEST, 6 MWT and, 10 MWT. Our findings show that these measures are not discriminate when used in the suspected NPH population.

When evaluating patients for possible NPH, the primary objective is to determine if there was a significant change in the patient’s symptoms following temporary drainage of CSF. Many different scales have attempted to quantify change following temporary CSF drainage. The vast majority of the scales are grading scales [7, 33–37]. Grading scales use categorical cutoffs to separate patients based on their level of impairment. After the temporary CSF drainage, if the patients’ Post-drain scores are within a less impaired category, they are considered improved. One commonly used rating scale for NPH was created using data from geriatric normative data. This scale assesses NPH using four separate domains (gait, neuropsychological, balance, and continence), all normalized to a 100-point scale. For a patient to be considered improved, they have to improve by greater than four points overall [7]. The limitation of this grading scale and others created for NPH is that it does not have an empirical method for differentiating chance improvement from a significant change at an individual patient level. The criteria for improvement in this grading scale is based on summed overall improvement across all four domains. This allows for change in score that is due to chance to contribute towards perceived improvement, thus to the recommendation for shunt surgery.

In this study, we attempted to address this issue by creating SRB clinically significant change models for common gait and cognitive measures (TUG, Dual TUG, 10 MWT, Mini-BEST, MoCA, SDMT, and 6 MWT) using data collected from patients presenting with suspected NPH that underwent temporary drainage of CSF. Incorporating clinical change model brings an empirical approach to the selection of individual patients for shunt surgery. Clinically significant change models allow physicians to make an evidenced based decision about the clinical significance of change following temporary drainage of CSF.

For temporary drains of CSF, the TUG was shown to be an efficient measure of gait for patients presenting with suspected NPH. The TUG does not require a trained physical therapist and can be administered by clinical staff in approximately 3 minutes. It is a reliable predictor of shunt outcome in NPH patients [38–40]. In our analysis, the TUG was significantly related to detailed measures of balance, endurance, and gait velocity.

This study has both strengths and limitations. Strengths include a large number of patients with detailed and well-established quantitative measures of several gait and cognitive measures. The high test-retest reliability of the measures allows for reliable clinical change models to be computed. Limitations include the unknown sensitivity and specificity of shunt outcomes for patients selected based on the clinical change models. To address this lack of information a study is planned to use the clinical change models in the center for CSF disorders to select patients for shunt surgery and assess both the short and long-term shunt outcome of the patients. As well, we intend to establish Minimal Clinically Important Difference models for shunted patients to assess the efficacy of shunting patients at both the individual and group levels.

## Conclusion

Standardized Regression Based Clinically Significant Change Models allow for physicians to use an evidenced based approach to differentiate clinically significant change from chance improvement at the individual patient level. The Timed Up & Go was shown to be predictive of detailed measures of gait velocity, balance, and endurance.

## Data Availability

Anonymized raw data will be made available upon reasonable request.

## Abbreviations

NPH: Normal Pressure Hydrocephalus
CSF: Cerebrospinal Fluid
EI: Evans index
10 MWT: 10 meter walk test
TUG: Timed Up & Go.
6MWT: 6-Minute Walk Test
Mini-BEST: Mini-Balance Evaluation Systems Test
MoCA: Montreal Cognitive Assessment
SDMT: Symbol Digit Modalities Test
